# Nutritional status of school children in the South Tongu District, Ghana

**DOI:** 10.1371/journal.pone.0269718

**Published:** 2022-08-24

**Authors:** Richard Gyan Aboagye, Nuworza Kugbey, Bright Opoku Ahinkorah, Abdul-Aziz Seidu, Abdul Cadri, Samuel Adolf Bosoka, Paa Yeboah Akonor, Mohammed Takase

**Affiliations:** 1 Fred N. Binka School of Public Health, University of Health and Allied Sciences, Hohoe, Ghana; 2 Department of General Studies, University of Environment and Sustainable Development, Somanya, Ghana; 3 Faculty of Health, School of Public Health, University of Technology Sydney, Sydney, Australia; 4 Centre for Gender and Advocacy, Takoradi Technical University, Takoradi, Ghana; 5 College of Public Health, Medical and Veterinary Sciences, James Cook University, Australia; 6 Department of Social and Behavioural Science, School of Public Health, University of Ghana, Legon- Accra, Ghana; 7 Department of Epidemiology and Biostatistics, Fred N. Binka School of Public Health, University of Health and Allied Sciences, Hohoe, Ghana; National Research Centre of Egypt, EGYPT

## Abstract

**Background:**

Malnutrition is a major public health problem because of the devastating consequences it has on children, their families, and society at large. Our study, therefore, sought to determine the prevalence of undernutrition and overweight/obesity and its associated factors among children aged 6–12 in the South Tongu District, Ghana.

**Methods:**

A school-based cross-sectional study was conducted among 423 school children aged 6–12 years in the South Tongu District of Ghana. A multistage sampling method was employed to recruit the school children for the study. A semi-structured questionnaire was used to collect data from the respondents. We used a dual-purpose (height and weight) measuring scale to obtain the anthropometric data. The World Health Organization’s AnthroPlus software was used to generate the z-scores for determining the nutritional status. Percentages were used to present the results of the prevalence of undernutrition and overweight/obesity among school children. Bivariate and multivariable binary logistic regression were used to examine the factors associated with undernutrition and overweight/obesity among school children. The results were presented as crude odds ratios (CORs) and adjusted odds ratios (AORs), with their 95% confidence interval (CI). Statistical significance was set at p<0.05. Stata 16.0 was used to perform the analyses.

**Results:**

The overall prevalence of undernutrition and overweight/obesity were 21.5% (CI = 17.7, 25.7) and 24.8% (CI = 20.8, 29.2), respectively. Specifically, the prevalence of stunting, thinness, underweight, overweight, and obesity were 10.4%, 12.1%, 3.8%, 11.1%, and 13.7%, respectively. School children whose household used water from non-portable sources were more likely to be undernourished [AOR = 2.03, 95% CI = 1.13, 3.63]. The odds of overweight/obesity was higher among school children whose mothers had attained formal education [AOR = 2.10, 95% CI = 1.09, 4.06], those who consumed beverages between meals per day [AOR = 1.87, CI = 1.08, 3.24], and those who had adequate dietary diversity score [AOR = 1.65, 95% CI = 1.02, 2.67]. School children aged 10–12 were less likely to be overweight/obese [AOR = 0.58, 95% CI = 0.35, 0.94] compared to those aged 6–9.

**Conclusion:**

The study showed a relatively high prevalence of undernutrition and overweight/obesity among school children in the South Tongu District. The identified risk factor(s) for undernutrition was the usage of water from non-potable sources whilst those of overweight/obesity were age (10–12 years), maternal formal education, beverage consumption between meals per day, and adequate dietary diversity. The findings reaffirm that malnutrition is still prevalent among school children. Hence, there is a need for the Ministry of Health, Ghana Education Service, and other Non-Governmental Organizations to pay critical attention to these factors to achieve the Sustainable Development Goal 2, target 2.2. Nutritional behavioural change education should be carried out among parents and school children. School health service activities should be intensified with a special focus on nutritional screening.

## Background

Malnutrition, defined as the deficiencies, excesses, or imbalances in an individual’s intake of energy and/or nutrients [1] is a major public health concern due to its devastating consequences on children, their family, and society [2,3]. Malnutrition is associated with at least one health complications, most especially among children in their developmental stages [4]. Global data on malnutrition indicators showed that about 144.0 million children were stunted, 47 million were wasted, 14.3 million were severely wasted, and 38.3 million were overweight or obese [5]. Compared to previous estimates [5], the present statistics show a decline in undernutrition whilst overweight is increasing tremendously resulting in the double burden of malnutrition, a term referred to as the co-existence of undernutrition and overweight/obesity [5,6]. However, the data presented above is only for children under the age of five, with minimal information available for children aged six to twelve, indicating a gap.

Undernutrition is the most prevalent form of malnutrition with stunting, thinness, and underweight as its indicators [7]. It has been reported that almost half of all under-five mortality cases were linked to undernutrition [5,8]. Out of the proportion of mortality, the largest burden was associated with the presence of infectious disease [9] with the severity confounded in children with a compromised immune system [10,11].

Sub-Saharan Africa continues to have the greatest rate of childhood mortality, with one out of every thirteen children dying before reaching the age of five due to nutrition-related causes [12]. Grantham-McGregor [13] posits that undernutrition in the early stages of life is associated with poor physical growth, cognitive, motor, and socio-emotional development, with a subsequent negative impact on their educational productivity. Studies conducted in parts of sub-Saharan Africa reported varied prevalence of undernutrition. For instance, a study in Northwest Ethiopia reported a 35.5% prevalence of undernutrition [14]. Also, Erismann et al. [15] in Burkina Faso and Teh et al. [16] in Cameroon found a 35.1% and 34.8% prevalence of undernutrition among children, respectively. Another study conducted in Cameroon reported that 22.8% of children were undernourished [17].

Similarly, childhood overweight/obesity is a major public health challenge in the 21^st^ century [18–20]. Overweight/obesity is prevalent in low- and middle-income countries despite the previous assertion of being widespread in high-income countries [18]. According to the World Health Organization (WHO) [21], overweight/obese children are at increased risk of type 2 diabetes, asthma, and hypertension. Also, overweight/obesity serves as a risk factor for other non-communicable diseases [21]. Likewise, overweight/obese children are more likely to become overweight/obese adults and experience its associated negative consequences [2]. A study conducted among children in Saudi Arabia showed the prevalence of overweight/obesity to be 29.6%. Studies conducted in China found a 20.0% [22] and 15.2% [23] prevalence of overweight/obesity among children. Other cross-sectional studies conducted in various parts of Africa reported the prevalence of overweight/obesity to be 14.7% in Eastern Ethiopia [24], 13.8% in Northwest Ethiopia [25], 11.9% in Northwest Ethiopia [26], 11.4% in Mozambique [27], 12.5% in Cameroon [28], and 3.0% in Nigeria [29].

In Ghana, the 2014 Demographic and Health Survey (DHS) report showed that 19%, 5%, 11%, and 3% of children under five years were stunted, wasted, underweight, and overweight respectively [30]. Researchers have reported the a varied prevalence of overweight/obesity among children of school-going age [31–39]. For instance, a study in Hohoe Municipality by Agbozo et al. [32] found that 9.3%, 8.5%, and 5.7% of school children were underweight, stunted, and thin. Kwabla et al. [37] in the La-Nkwantanang Municipality reported that 16.7% and 6.7% of children in basic schools were stunted and thin, respectively.

Evidence suggests that socio-demographic characteristics such as age [25,40], sex [22,37,41], geographical area [32,42], type of school [31,32,43], parents’ educational level [14,22,25,44,45], parents’ employment status [45,46], socioeconomic status [22,24,26,28,31], and dietary diversity [25,47,48] have been linked to malnutrition in children. Other researchers posit that infections or illness [15], household usage of non-potable water, and poor hygiene and sanitary practices [49–51] were factors associated with undernutrition. On the other hand, genetics [52], imbalances between calories consumed and calories expended [52], medical conditions [52], sedentary lifestyles [24,26,31,45], and consumption of fast and energy-dense foods [45,53] were the risk factors for overweight/obesity in children.

School-going age is a dynamic period characterized by physical growth and mental development [54,55]. As a result, nutritional deficits during this period can subject the child to adverse consequences such as low school enrolment, early dropout, scholastic backwardness, and reduced work capacity [54]. Despite the numerous nutritional status assessment studies conducted in Ghana, much focus has been on preschoolers (5 years and below) to the neglect of children aged 6 years and above. Also, data from the South Tongu District Health Directorate highlighted that undernutrition indicators are still prevalent among children under five years but no data on those above five years (Annual Report (Unpublished), 2020). Additionally, unlike the regular nutritional status assessment done for children below five years, there is no specific interventional assessment aimed at those above 6 years. Hence, there is a need to undertake the current study to ascertain the district-specific factors associated with the nutritional status of the school children with a focus on those aged 6–12 years mostly in basic schools in the South Tongu District. The study’s contribution is that it provides policymakers, healthcare practitioners, and other key stakeholders with valuable information for planning and implementing programs and interventions targeted at improving school children’s nutritional status in order to improve their health and wellbeing. This research also adds to the current body of knowledge on malnutrition and its related factors in school-aged children.

## Methods

### Study setting

We conducted the study in the South Tongu District, one of the 17 administrative Municipalities/Districts in the Volta Region. The district lies between latitudes 6°10’ and 5°45’ North and longitudes 30°30’ and 0°45’ East. The district is located in the southern part of the Lower Volta Basin and bounded to the north by the Central and North Tongu Districts, to the east by the Akatsi South District, to the west by the Ada East District of the Greater Accra Region, and to the south by the Keta Municipality [56]. The district has an estimated population of 87,950 of which 21,937 are aged 5–14. Majority of the inhabitants are females (54.5%). The population density of the district is 136.7 persons per square kilometer. The inhabitants in the district are involved in cash crop farming, subsistence farming, livestock rearing, and fishing [56]. The district has six (6) sub-districts and 154 basic schools of which 111 and 43 were public and private schools respectively [(Annual Report (Unpublished), 2020)]. The district was selected for the study because of the high prevalence of undernutrition indicators (stunted [22.0%], underweight [21.5%], and wasting [8.4%]) among children [57].

### Study design and population

We employed a school-based cross-sectional design which was carried out descriptively among the school children. The study population comprised school children aged 6–12 years from registered basic schools in the South Tongu District. We included children aged 6–12 in primary 1–6. School children with a known medical condition and those who were sick were excluded from the study. We relied on strengthening the reporting of observational studies in epidemiology guidelines in writing the manuscript (S1 Table) [58].

### Sample size determination

We calculated the sample size for the study using the formulae; n = $\frac{\left(Z\alpha_{/2)2}P\;(1‐P\right)}{e^{2}}$ [59]. Where n = sample size; Z_α/2_ = Z-score of 1.96 at 95% confidence interval (CI); p = prevalence or proportion of malnutrition indicators from a previous study; and e = margin of error of 5.0%. With a 50.3% prevalence of stunting among school children in Nkwanta South, Ghana [60], and a 10% non-response rate, a sample size of 423 school children aged 6–12 was estimated for the study.

### Sampling procedure

A multistage sampling procedure was used to recruit 423 school children for the study.

#### First stage

At the initial stage, we obtained an official list containing 154 basic schools in the district from the South Tongu District Education Office. We grouped the schools into public feeding, public non-feeding, and private basic schools. All the schools whose population consisted of preschoolers were dropped. A simple random sampling method using a balloting technique was used to select three schools from each of the three groups. We used a total of six basic schools for the study.

#### Second stage

At the second stage, a stratified sampling method was used to apportion the sample size for a selected school based on the number of children aged 6–12. Thus, each of the six schools was treated as a separate stratum. The sample size for a selected school was estimated as the product of the total number of children aged 6–12 years in a selected school and the total sample size for the study, all divided by the total number of children aged 6–12 years in all six schools.

#### Stage three

The third stage involved the selection of respondents at the school level. At the school level, each of the classes was further treated as a stratum. The number of children from each class in a school was representative of the number of children aged 6–12 in that class considering the sample size for the selected school.

#### Fourth stage

At the final stage, respondents were selected from the classroom level. Based on the number of children aged 6–12 years in the classroom, and the number of children to select from each school, the specific number of children to select per each classroom was proportional to the class size aged 6–12 years. Afterward, a simple random sampling method using the lottery method was used to recruit the children based on the required sample size for a particular class in a selected school. Pieces of paper with the inscription “YES” or “NO” on them were placed in a container and thoroughly shaken. Any child who picked “YES” was included in the study, subject to parental or guardian consent and the child’s assent. We repeated the procedure in all schools until we obtained the required sample size.

**Distribution of respondents**

**Table:** 

**Name of schools**	**Total population**	**Sample chosen**
**Public feeding school**		
Sogakope EP Basic	427	67
Sokpoe Presby Basic	536	84
**Public Non-feeding school**		
Sogasco Basic	434	68
Sogakope Presby Basic	472	74
**Private school**		
Little star Academy	472	74
Lower Volta Academy	357	56
**Total**	**2698**	**423**

Source: Field work, 2019.

### Data collection procedure

The school children aged 6–12 from the selected schools who met the inclusion criteria were presented with parental/guardian consent forms for the approval of parents/guardians and child assent forms for the child’s voluntary approval. School children who returned both signed written parental or guardian consent forms and assent forms, identified by their respective codes and names in an excel document, were interviewed together with their parent/guardian or teacher. A semi-structured interviewer-administered questionnaire was used to collect data from the school children. The data collection took place at the selected schools of the children together with their parent/guardian or a teacher. A questionnaire was used to collect data such as socio-demographic characteristics of the child, feeding practices, physical activity level, sources of water for domestic use, and handwashing practices. The questionnaires were completed by the child and their parents/guardians. Anthropometric measurements (height and weight) were done using a dual-purpose measuring scale. The instrument is attached as a supplementary file (S2 Table). Five (5) Community Health Nurses (CHNs) were trained and assisted in the data collection. We trained the CHNs for three days covering issues such as proper filling and completeness of questionnaire, usage of measurement scale, and a proper reading of the measuring scale to reduce inter-and intra-observer errors.

### Anthropometric measurement

Anthropometric measurements (weight and height) were taken using standard procedures [52]. We measured weight and height using an electronic weight and height scale, a dual-purpose device for measuring weight and height with model number: TCS 200LP. Height and weight were measured to the nearest 0.1cm and 0.1kg respectively. Upon taking the measurement, we asked the children to remove their shoes and any heavy clothing except their school clothes. We made each respondent stand on the scale without holding onto any support with feet closed, hands by the sides, and head in a forward position. The top of the height board was placed on top of the head of the child and then the reading was taken at right angles to the board behind the child (wall). Height and weight were then read. The measurements were taken twice and the average was used for analysis. The accuracy of the measuring device was checked daily and the test for intra-observer and inter-observer reliability was conducted before the onset of data collection. The device was charged after every 2 hours. The starting readings were adjusted to zero before any measurement was taken.

### Study variables

#### Dependent variables

The dependent variables were undernutrition and overweight/obesity. The indicators of undernutrition comprised of underweight (Weight-for-Age Z-score [WAZ]), stunting (Height-for-Age Z-score [HAZ]), and thinness or wasting (Body Mass Index-for-Age Z-score [BMIZ]) [52]. Any child with one or more of the undernutrition indicators was classified as undernourished. Overweight and obesity are BMI-for-age Z-score (BAZ). We categorized any child having either overweight or obesity or both as overweight/obese. The dependent variables were determined independently.

#### Independent variables

Independent variables in the study comprised socio-demographic characteristics, feeding practices, sedentary lifestyle, source of water for domestic activities, and handwashing practices. The socio-demographic variables include sex (male/female), age (6-9/10-12), type of school (public feeding/public non-feeding/private schools), area of residence (rural/urban), religion (Christian/non-Christian), class (lower primary [class 1–3]/upper primary [class 4–6]), marital status of parents (single/married), parents’ educational status (formally educated/not educated), and employment status of parents (employed/unemployed). We assessed a sedentary lifestyle using physical activity. We dichotomized physical activity as (yes/no).

Dietary practices assessed included consumption of breakfast (yes/no), daily food intake (less than three/three or more), consumption of sweets and beverages in-between meals (yes/no), and dietary diversity (adequate/inadequate). Dietary diversity score (DDS) was qualitatively determined using the food eaten by the child within 24 hours before the data collection based on the recommended seven food groups [61]. The food groups comprised; (i) grains, roots and tubers; (ii) legumes and nuts; (iii) dairy products; (iv) flesh foods (meats/fish/poultry); (v) eggs; (vi) vitamin A-rich fruits and vegetables; and (vii) other fruits and vegetables. We asked the school children to recall all food items and beverages consumed within 24 hours. The scoring was done by counting the number of food groups the child had consumed in the last 24-hours before the data collection. The dietary diversity score ranged from 0 to 7 with a minimum of 0 if none of the food groups was consumed and 7 if they consumed all the food groups. We said a child to have an adequate dietary diversity score if the child consumed at least four different food groups (DDS ≥ 4) [62].

We classified the sources of water for domestic activities in various households of school children into potable or non-potable sources based on the WHO’s classification [51]. Households whose source of water was either piped, borehole, or projected wells were grouped into potable sources whilst non-potable sources comprised unprotected well, rainwater collection, rivers, and streams [51].

Handwashing practices among the children were determined using the mode (handwashing with soap and water, with water only, with ash, and no handwashing) and frequency of washing hands (before eating, after eating, after playing, and after defecation). School children whose mode of handwashing was either with soap and running water only or with ash and running water; and frequently washed their hands before eating, after eating, after playing, and after using the washroom were classified as engaging in good handwashing practice as determined through factor analysis. We classified any other response besides the stated criteria as poor handwashing practice [51,62] (S2 Table).

### Data quality control

Five CHNs were trained and assisted with the data collection. We trained the CHNs for three days covering issues such as proper filling and completeness of questionnaire, usage of measurement scale, and a proper reading of the measuring scale to reduce inter-and intra-observer errors. A standardization test was carried out on school children who were not selected to include in the study, and the results were used to correct any error before actual data collection took place. We pretested the study questionnaire on 10.0% of the study’s sample size outside the randomly selected schools and the results were used to modify the questionnaire for final data collection.

### Statistical analyses

The collected data were assigned unique codes before we entered the data using Epi-Data software version 3.1. The entered data were later exported to Stata version 16.0 for analysis. We represented data using tables and graphs. Continuous variables were reported as mean ± standard deviation and we reported categorical variables as proportions. Height and weight values, age in complete years, and sex were exported to WHO AnthroPlus software which has the Growth Reference Standard for children and adolescents 5–19 years [63] incorporated in it to generate Z-scores. The resulting z-scores were exported back to Stata software to determine the proportion of children who were stunted, underweight, wasted/thin, overweight, and obese. The growth reference classification of the nutritional indicators consists of stunting (HAZ <-2SD), underweight (WAZ <-2SD), thinness (BAZ <-2SD), overweight (BAZ >+1SD to <+2SD) and obesity (BAZ >+2SD) [52,63]. However, underweight was not calculated for children above 10 years because of an increase in hormonal growth in children above 10 years [52,64]. Both descriptive and inferential analyses were performed. The descriptive analysis was conducted using frequencies and percentages and the results were presented using tables and a graph. The inferential analysis was carried out using binary logistics regression. We performed a bivariate and multivariable binary logistic regression analysis to examine the factors associated with undernutrition and overweight/obesity. The results were presented in a tabular form showing the crude odds ratios (CORs) and adjusted odds ratios (AORs) with their accompanying 95% confidence intervals (CIs). A p<0.05 was considered statistically significant. Additionally, a multicollinearity test using the variance inflation factor (VIF) was conducted to ascertain the existence of collinearity among the variables. The results showed that the minimum, maximum, and mean VIF were 1.04, 1.80, and 1.22, respectively. Hence, there was no evidence of collinearity among the variables used in the study.

### Ethical consideration

We obtained ethical approval from the University of Health and Allied Sciences Research Ethics Committee (UHAS-REC) with approval number UHAS-REC.A.6 [4] 18–19. Before the commencement of the study, we sought institutional permission from the South Tongu District Health Directorate and the District Education Service. We also obtained written parental/guardian consent from each of the children before we included them in the study. A detailed explanation of the procedures and processes involved in the study were given to the school authorities and subsequently to the school children and their parents or guardians. The school children were allowed to voluntarily assent to participate or not. However, only school children aged 6–12 years who voluntarily assented and parents consented were included in the study. We sought permission from the Heads of the various schools and the respondents before data collection. With the help of the CHNs, severely malnourished children were referred to health facilities for treatment and rehabilitation.

## Results

### Sociodemographic and background characteristics of the school children

Table 1 shows the sociodemographic and background characteristics of the children. It was found that the majority (58.6%) of the children were aged 6–9 years. The mean age of the children was 9.0 ± 1.9 years. More than half (52.5%) of the respondents were females. A little over 91% were Christians. One hundred and fifty-one (35.7%) of the children attended public schools with a feeding programme, followed by the public school without a feeding programme (33.6%) with only 30.7% from private schools. Most (69.3%) of the children were in the lower primary (class 1–3). Most of the children had their mothers formally educated (76.8), unemployed mothers (80.9%), formally educated fathers (82.3%), and unemployed fathers (61.5%). The majority (64.5%) of the children took breakfast every morning. Almost all (91.7%) of the children had good daily food intake (ate at least times per day). Most (67.4%) of the children took beverages between meals per day. Almost 33% of the children practiced adequate dietary diversity. The majority of 68.3% of the children performed physical activity. The vast majority (84.2%) of the children were from homes that had access to potable water for domestic activities. Two hundred and sixty-five (62.6%) of the children practiced good handwashing.

**Table 1:** Sociodemographic and background characteristics of the respondents (n = 423).

Variables	Frequency (n = 423)	Percentage (%)
Mean age (years ± SD)	**9.0 ± 1.9 years**	
**Age group (years)**		
6–9 years	248	58.6
10–12 years	175	41.4
**Sex**		
Female	222	47.5
Male	201	52.5
**Religion**		
Christian	386	91.3
Non-Christian	37	8.7
**Place of residence**		
Rural	293	69.3
Urban	130	30.7
**Class**		
Lower Primary (Class 1–3)	293	69.3
Upper Primary (Class 4–6)	130	30.7
**Type of school**		
Public feeding	151	35.7
Public non-feeding	142	33.6
Private	130	30.7
**Mother’s educational status**		
Non-formal	98	23.2
Formal	325	76.8
**Mother’s employment status**		
Unemployed	342	80.9
Employed	81	19.1
**Mother’s marital status**		
Single	72	17.0
married	351	83.0
**Father’s educational status**		
Non-formal	75	17.7
Formal	348	82.3
**Father’s employment status**		
Unemployed	260	61.5
Employed	163	38.5
**Breakfast consumption**		
Yes	273	64.5
No	150	35.5
**Daily food intake**		
Poor (<3) times	35	8.3
Good (≥3) times	388	91.7
**Beverage intake between meals per day**		
Yes	138	32.6
No	285	67.4
**Dietary diversity score (DDS)**		
Inadequate (DDS <4)	284	67.1
Adequate (DDS ≥4)	139	32.9
**Physical activity**		
Yes	289	68.3
No	134	31.7
**Source of water**		
Potable	356	84.2
Non-potable	67	15.8
**Hand washing practice**		
Good	265	62.6
Bad	158	37.4

### Nutritional status of school children

**Fig 1:**
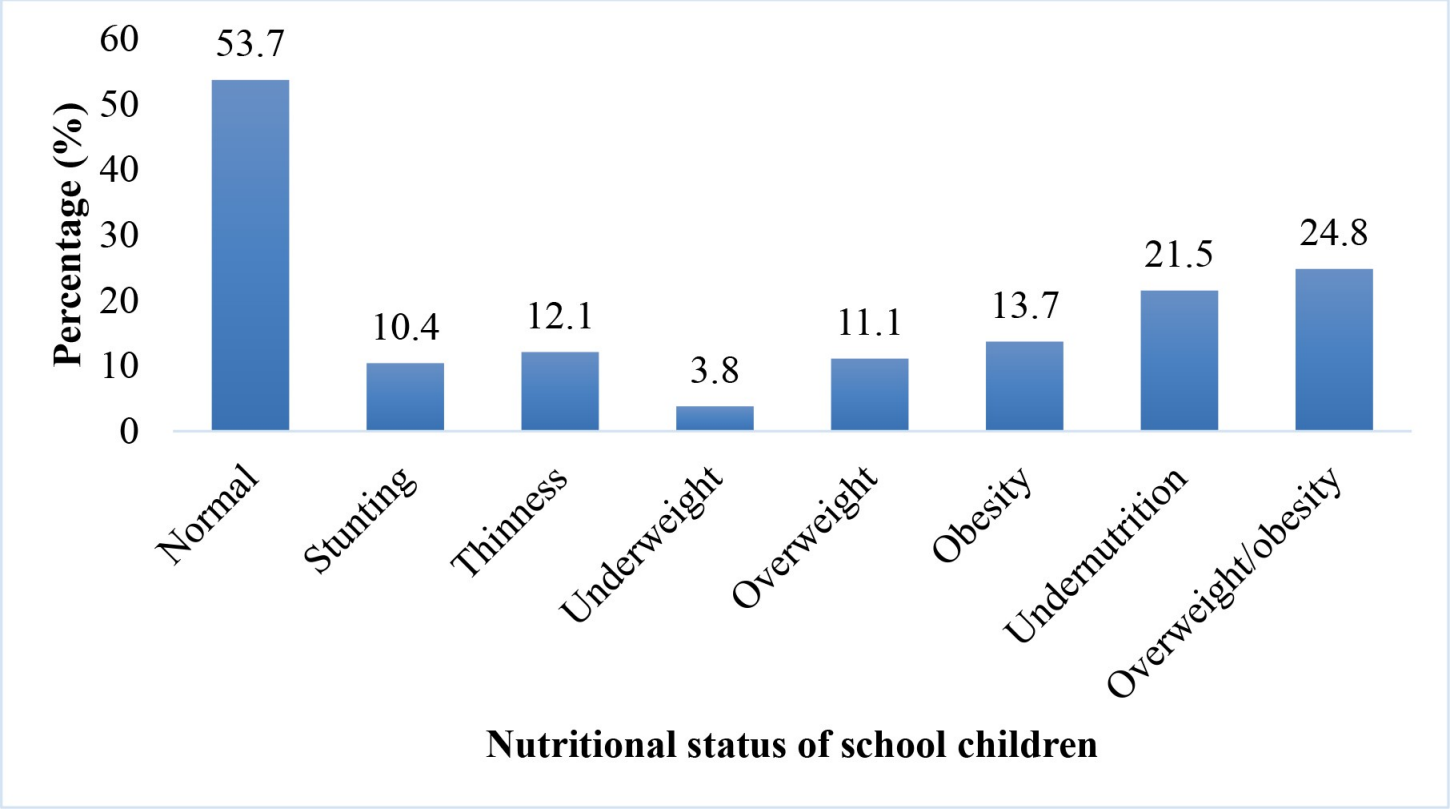
Nutritional status of school children aged 6–12.

The overall prevalence of undernutrition among the school children was 21.5% (95% CI = 17.7–25.7). Out of this, the prevalence of undernutrition indicators were stunting (10.4%), underweight (3.8%), and thinness (12.1%). Also, the prevalence of overweight/obesity was 24.8% (95% CI = 20.8–29.2) of which the prevalence of overweight and obesity were 11.1% and 13.7%, respectively (Fig 1).

### Factors associated with undernutrition and overweight/obese among school children 6–12 years

Results from the multivariable logistic regression model in Table 2 showed that only the source of water was significantly associated with undernutrition after controlling for confounders. Children whose households used water from non-potable sources were more likely to be undernourished [AOR = 2.03, 95% CI = 1.13, 3.63].

**Table 2:** Bivariate and multivariable regression analysis of factors associated with undernutrition.

Variables	Nutritional status			
	Normal n (%)	Undernourished n (%)	COR (95% CI)	AOR (95% CI)
**Age group (years)**				
6–9 years	195 (78.6)	53 (21.4)	1.0	1.0
10–12 years	137 (79.2)	38 (20.8)	1.02 (0.64, 1.63)	1.12 (0.69, 1.82)
**Sex**				
Male	152 (75.6)	42 (24.4)	1.0	1.0
Female	180 (81.1)	49 (18.9)	0.72 (0.45, 1.15)	0.81 (0.50, 1.31)
**Religion**				
Christian	306 (79.3)	80 (20.7)	1.0	
Non-Christian	26 (70.3)	11 (29.7)	1.62 (0.77, 3.41)	
**Place of residence**				
Rural	228 (77.8)	65 (22.2)	1.0	
Urban	104 (80.0)	26 (20.0)	0.88 (0.53, 1.46)	
**Class**				
Lower primary (Class 1–3)	231 (78.8)	62 (21.2)	1.0	
Upper primary (Class 4–6)	101 (77.7)	29 (22.3)	1.07 (0.65, 1.76)	
**Type of school**				
Public feeding	120 (79.5)	31 (20.5)	1.0	
Public non-feeding	109 (76.8)	33 (23.2)	1.17 (0.67, 2.04)	
Private	103 (79.2)	27 (20.8)	1.01 (0.57, 1.81)	
**Mothers educational status**				
Non-formal	78 (79.6)	20 (20.4)	1.0	
Formal	254 (78.2)	71 (21.8)	1.09 (0.62, 1.90)	
**Mothers employment status**				
Unemployed	267 (78.1)	75 (21.9)	1.0	
Employed	65 (80.2)	16 (19.8)	0.88 (0.48, 1.60)	
**Marital status**				
Single	60 (83.3)	12 (16.7)	1.0	
Married	272 (77.5)	79 (22.5)	1.45 (0.74, 2.83)	
**Fathers educational status**				
Non-formal	50 (66.7)	25 (33.3)	1.0	1.0
Formal	282 (81.0)	66 (19.0)	0.47** (0.27, 0.81)	0.57 (0.32, 1.02)
**Fathers employment status**				
Unemployed	194 (74.6)	66 (25.4)	1.0	1.0
Employed	138 (84.7)	25 (15.3)	0.53** (0.32, 0.89)	0.66 (0.39, 1.14)
**Breakfast consumption**				
Yes	213 (78.0)	60 (22.0)	1.0	
No	119 (79.3)	31 (20.7)	0.92 (0.57, 1.51)	
**Daily food intake**				
Poor (<3) times	30 (85.7)	5 (14.3)	1.0	
Good (≥3) times	302 (77.8)	86 (22.2)	1.71 (0.64, 4.54)	
**Beverage intake between meals per day**				
No	103 (74.6)	35 (25.4)	1.0	
Yes	229 (80.4)	56 (19.6)	0.72 (0.44, 1.17)	
**Dietary diversity score (DDS)**				
Inadequate (DDS <4)	226 (79.6)	58 (63.7)	1.0	
Adequate (DDS ≥4)	106 (76.3)	33 (23.7)	1.21 (0.75, 1.97)	
**Source of water**				
Potable	288 (80.9)	68 (19.1)	1.0	1.0
Non-potable	44 (65.7)	23 (34.3)	2.21** (1.25, 3.91)	2.03* (1.13, 3.63)
**Handwashing practice**				
Bad	119 (75.3)	39 (24.7)	1.0	
Good	213 (80.4)	52 (19.6)	0.74 (0.46, 1.19)	

COR = Crude Odds Ratio; AOR = Adjusted Odds Ratio, 1 = Reference

*p<0.05

**p<0.01.

Similarly, children aged 10–12 were 42% less likely to be overweight/obese compared to those aged 6–9 years [AOR = 0.58, 95% CI = 0.35, 0.94]. Children whose mothers were formally educated had higher odds of being overweight/obese [AOR = 2.10, 95% CI = 1.09, 4.06]. Also, children who consumed beverages between meals per day were more likely to be overweight/obese [AOR = 1.87, 95% CI = 1.08, 3.24]. Children who had adequate dietary diversity scores were more likely to be overweight/obese [AOR = 1.65, 95% CI = 1.02, 2.67] (See Table 3).

**Table 3:** Bivariate and multivariable regression analysis of factors associated with overweight/obesity.

	Nutritional status			
Variable	Normal N (%)	Overweight/obesity n (%)	COR (95% CI)	AOR (95% CI)
**Age group (years)**				
6–9 years	176 (55.3)	72 (68.6)	1.0	1.0
10–12 years	142 (44.7)	33 (31.4)	0.57* (0.36, 0.91)	0.58* (0.35, 0.94)
**Sex**				
Male	156 (49.1)	45 (45.9)	1.0	1.0
Female	162 (50.9)	60 (57.1)	1.28 (0.82, 2.00)	1.26 (0.78, 2.02)
**Religion**				
Christian	293 (92.1)	93 (88.6)	1.0	
Non-Christian	25 (7.9)	12 (11.4)	1.51 (0.73, 3.13)	
**Place of residence**				
Rural	220 (69.2)	73 (69.5)	1.0	
Urban	98 (30.8)	32 (30.5)	0.98 (0.61, 1.59)	
**Class**				
Lower primary (Class 1–3)	215 (67.6)	78 (74.3)	1.0	
Upper primary (Class 4–6)	103 (32.4)	27 (25.7)	0.72 (0.44, 1.19)	
**Type of school**				
Public feeding	105 (33.0)	46 (43.8)	1.0	1.0
Public non-feeding	114 (35.9)	28 (26.7)	0.56* (0.33, 0.96)	0.70 (0.40, 1.23)
Private	99 (31.1)	31 (29.5)	0.71 (0.42, 1.22)	0.58 (0.33, 1.04)
**Mother’s educational status**				
Non-formal	84 (26.4)	14 (13.3)	1.0	1.0
Formal	234 (73.6)	91 (86.7)	2.33** (1.26, 4.32)	2.10* (1.09, 4.06)
**Mother’s employment status**				
Unemployed	266 (83.6)	76 (72.4)	1.0	1.0
Employed	52 (16.4)	29 (27.6)	1.95* (1.16, 3.29)	1.37 (0.78, 2.43)
**Marital status**				
Single	51 (16.0)	21 (20.0)	1.0	
Married	267 (84.0)	84 (80.0)	0.76 (0.43, 1.34)	
**Father’s educational status**				
Non-formal	63 (19.8)	12 (11.4)	1.0	
Formal	255 (80.2)	93 (88.6)	1.91 (0.99, 3.71)	
**Father’s employment status**				
Unemployed	208 (65.4)	52 (49.5)	1.0	1.0
Employed	110 (34.6)	53 (50.5)	1.93** (1.23, 3.01)	1.59 (0.99, 2.55)
**Breakfast consumption**				
Yes	205 (64.5)	68 (64.8)	1.0	
No	113 (35.5)	37 (35.2)	0.99 (0.622, 1.57)	
**Daily food intake**				
Poor (<3) times	26 (8.2)	9 (8.6)	1.0	
Good (≥3) times	292 (91.8)	96 (91.4)	0.95 (0.43, 2.10)	
**Eat between meals per day**				
No	116 (36.5)	22 (21.0)	1.0	1.0
Yes	202 (63.5)	83 (79.0)	2.17** (1.28, 3.65)	1.87* (1.08, 3.24)
**Dietary diversity score (DDS)**				
Inadequate (DDS <4)	224 (70.4)	60 (57.1)	1.0	1.0
Adequate (DDS ≥4)	94 (29.6)	45 (42.9)	1.79* (1.13, 2.82)	1.65* (1.02, 2.67)
**Physical activity**				
Yes	225 (70.8)	64 (61.0)	1.0	
No	93 (29.2)	41 (39.0)	1.55 (0.98, 2.46) 0.062	

COR = Crude Odds Ratio; AOR = Adjusted Odds Ratio, 1 = Reference

*p<0.05

**p<0.01.

## Discussion

This study examined the nutritional status and its associated factors among school-going children aged 6–12 in the South Tongu District. The prevalence of undernutrition among school children aged 6–12 in the current study was 21.5%, of which 12.1%, 10.4%, and 3.8% were thin, stunted, and underweight, respectively. The prevalence of undernutrition is similar to results obtained in Cameroon (22.8%) [17] and Ethiopia (21.2%) [65]. However, a high prevalence was reported in studies conducted in Nepal [66], Madagascar [67], Ethiopia [14,68,69], Kenya [70], and Burkina Faso [15]. For instance, a study in Nepal found a 26% prevalence of undernutrition [66]. Also, a 35.1% prevalence of undernutrition was found in Burkina Faso [15]. Socio-economic disparities, levels of household food insecurity, and differences in study periods could have played a significant role in the observed differences [71,72].

Undernutrition in the present study was higher compared to findings from studies in Cameroon and Nigeria. In Cameroon, Tabi et al. [73] (2019) found a 9.25% prevalence of undernutrition in primary school children. On the other hand, Umeokonkwo et al. [7] reported that 15.7% of school-aged children were undernourished in Nigeria. In explaining the observed discrepancy, we hypothesized that the prevalence in the study could be a result of the proximity of the district to Volta Lake, Ghana. The prevalence of parasitic infections, water-borne diseases, and water-related diseases found in towns along river bodies could have contributed to the high prevalence of undernutrition in our study [15,57]. The results further imply a negative effect on the physical growth and mental development of the school children as the age group 6–12 years marks also a period of growth transformation in the lives of children.

We found the prevalence of overweight/obesity to be 24.8%. Out of this, 11.1% and 13.7% were overweight and obese, respectively. This was similar to findings from a study that reported 22.6% and 22.9% prevalence of overweight andobesity among school children in Tanzania [40]. The observed prevalence of overweight/obesity was lower than the reported findings from Palestine. Among children aged 6–12 years, 14.5% were overweight whiles 15.7% were obese in Palestine [74]. The discrepancy in the findingscould be due to the use of the Center for Disease Control Classification of BMI [36] and the inclusion of children older than 12 years [35,36] in those studies compared to the present study, which utilised the WHO classification.

Overweight/obesity finding in the our study was higher than the 15.2% prevalence of overweight/obesity found in China [23]. The prevalence of overweight/obesity found in our study is also relatively higher than the 11.9% reported in children aged 6–12 years in Ethiopia [26], and 4.9% among children from Nigeria [43]. Increasing consumption of fatty and calorie-dense foods coupled with growing sedentary behaviors such as long hours watching television, playing computer games, and usage of energy-saving devices could have accounted for the differences in prevalence [75].

School children aged 10–12 years had lower odds of overweight/obesity in the present study. Decreasing overweight/obesity with increasing age was found in a study conducted in Tanzania among children [40]. Also, a study among school-age children in Palestine reported consistent findings [74]. Contrary to the findings in this study, Liu et al. [22] found overweight/obesity to increase with increasing age. The extent of physical activity exhibited by the children in this age group such as engaging in sporting activities and and the use a passive form of transport to school could have accounted for the association found in our study. This could justify the low prevalence of overweight/obesity (31.4%) in the children aged 10–12 in the current study.

Also, mothers’ formal education was found in the present study as a risk factor for overweight/obesity among school children. Similar findings were reported in studies conducted in China [22,76], Bahrain [77], Nepal [45], and Kenya [78]. Educated mothers are likely to be employed and subsequently gain higher income [41]. As a result, their children are often provided with energy-dense foods, and electronic devices to play with that promote sedentary lifestyles and hinder physical activity [41,78]. Also, overweight/obesity is sometimes perceived as good health and good living with limited knowledge of its implications in most wealthy homes [79]. This in turn promotes overweight/obesity in children whose mothers are educated.

Consumption of beverages between meals per day was a predictor of overweight/obesity in children. Several studies have shown consistent findings among children [36,80,81]. Sugar-sweetened beverages, fizzy drinks, and pastries consumed by children are mostly calories dense which could predispose a child to become overweight/obese in the absence of physical activity, the reason for the associated finding in our study [36,81].

The odds of overweight/obesity was higher among children who had adequate dietary diversity score compared to those with inadequate dietary diversity. Our finding is supported by results from other studies in South Asia [82], Northwest Ethiopia [25,48], and South Ethiopia [83]. These studies found a higher likelihood of overweight/obesity among school children with high dietary diversity. Despite the diversification of food groups, most children may heavily consume energy-dense foods with minimal intake of other food groups thereby increasing their propensity of becoming overweight/obese. Reduced physical activity by the school children could have increased their predisposition to overweight/obesity [53].

School children whose household used water from non-potable sources had higher odds of undernutrition. Consistent findings have been reported in several countries across the globe. For instance, studies in Iran [84], Ethiopia [85,86], Nigeria [87], and Tanzania [50,88] have all reported similar findings which correspond with that of the present study. Also, usage of safer water such as piped water was associated with lower odds of wasting in a study conducted in seven countries in sub-Saharan Africa [89]. However, such an association was not found in data from Niger and Lesotho [89]. Household usage of water for domestic activities such as drinking and cooking could have exposed the children to contracting water-borne and water-related diseases as well as helminthic infections, which impact poorly on the nutritional status of children [15]. Studies have shown that the provision of potable water could reduce the occurrence of diarrhea and soil-transmitted helminthic infections which have bearings on undernutrition among children [51,90]. This implies that the supply of potable water for household activities is key to promoting good health and alleviating undernutrition among children.

### Limitations of the study

The cross-sectional nature of the study was not appropriate to establish a causal relationship between the dependent and independent variables. Potential recall bias by the respondents in responding to questions on dietary practices and sedentary lifestyles could have impacted the study’s outcome. The qualitative nature of scoring the dietary diversity without quantifying the nutrient value of the foods the respondents consumed could have affected the results. Self-reporting of the handwashing practices and source of water for domestic use instead of actual demonstration and observation could have influenced the outcome of the study. Also, the study did not assess intestinal parasites and anaemia status which influence undernutrition. Moreover, data on the issues such as the share of the stomach, proportion, and types of unhealthy foods consumed weekly were not assessed, and this could have impacted on the study’s results. Additionally, sample weights were not applied in the current study, and this limits the generalizability of the findings to school children in South Tongu District.

## Conclusion

The study found a co-existence of undernutrition and overweight/obesity among the school children aged 6–12 in the South Tongu District. High prevalence of undernutrition (21.5%) and overweight/obesity (24.8%) were found in the school children. Overweight/obesity was associated with increasing age (10–12 years), maternal formal education, beverage consumption between meals per day, and adequate dietary diversity. Household usage of water from non-potable sources was associated with undernutrition. Given this, nutritional behavioural change education should be carried out among parents and school children. School health service activities should be intensified with a special focus on nutritional screening. Adequate provision of potable water supply should be made accessible. Further research should focus on the influence of household sources of water and dietary patterns on the nutritional status of children.

## Supporting information

S1 Table STROBE checklist. (DOCX)

S2 Table Study questionnaire. (DOCX)

S1 Data (XLS)

## Abbreviations

AOR: Adjusted Odds Ratio
BAZ: BMI-for-Age Z-score
BMI: Body Mass Index
BMIZ: Body Mass Index for Age Z-score
CI: Confidence Interval
CHNs: Community Health Nurses
COR: Crude Odds Ratio
DDS: Dietary Diversity Score
DHS: Demographic and Health Survey
HAZ: Height-for-Age Z-score
SD: Standard Deviation
UHAS-REC: University of Health and Allied Sciences Research Ethics Committee
UNICEF: United Nations Children’s Fund
WAZ: Weight-for-Age-Z-score
WHO: World Health Organization
