# Supplementary material for: Nutritional status of school children in the South Tongu District, Ghana
Source: PLoS One. 2022 Aug 24;17(8):e0269718. doi: 10.1371/journal.pone.0269718 (PMC9401153; doi:10.1371/journal.pone.0269718)
Supplement: S2 Table — (DOCX) [file pone.0269718.s002.docx]

**Nutritional status of school children in the South Tongu District, Ghana: Analysis of prevalence and associated factors from a cross-sectional study**

Participant code Date: …………………….

School: ……………………….

**Section I: Child’s Demographic Information**

| **S/N** | **Questions** | **Responses** | **Skip to** |
| --- | --- | --- | --- |
| 001 | What is your age in complete years | ………………….. |  |
| 002 | What is your date of birth (dd/mm/yyyy) |  |  |
| 003 | What is your sex? | 1=Male  2=Female |  |
| 004 | What is your ethnicity? | 1= Ewe  2= Akan  3= Northerner  4=Guan  5=Ga/Dangme  6=Other……. |  |
| 005 | What is your religion? | 1=Christian  2=Muslim  3=Traditionalist  4=Other ……... |  |
| 006 | Area of residence | …………………….. |  |
| 007 | Residence status | 1=Rural  2=Urban |  |
| 008 | Type of school | 1.Public school feeding  2. Public non-school feeding  3. Private school |  |
| 009 | Do you partake in school feeding programme? | 1 = Yes  2 = No |  |
| 010 | Which class are you? | Class……………. |  |
| 011 | Do you take money to school? | 1=Yes  2=No |  |
| 012 | If yes, how much do you take to school | GHC…………. |  |
| 013 | Who do you live with? | 1. Both parents 2. Mother 3. Father  4. Guardian |  |
| 014 | How many children are in the household? | …………………  Under five years………..  Between 6-12 years …… |  |

|  | **Section II: Child’s Feeding practices** | | |
| --- | --- | --- | --- |
|  | **Questions** | **Responses** | **Skip** |
| 015 | Do you eat breakfast at home every morning? | 1=Yes  2=No |  |
| 016 | Do you buy food at school to eat? | 1=Yes  2=No |  |
| 017 | If Yes, how often per day? | 1=Once  2=Twice  3=Three times |  |
| 018 | How many times do you eat in a day? | ……………………….. |  |
| 019 | How often do you take snacks in a day? | 1=None  2=Once  3=Twice  4=Three times and above |  |
| 020 | How often do you eat sweets per a day? | 1=None  2=Once  3=Twice  4=Three times and above |  |
| 021 | How often do you eat fruits per a day? | 1=None  2=Once  3=Twice  4=Three times  5=Four times and above |  |
|  | **Section III: Physical activity** | |  |
| 022 | Do you do any sporting activity? | 1=Yes  2=No | If no, go to (026) |
| 023 | If yes, which sporting activity? | 1=Football  2=Athletics  3=Ampe  4=others |  |
| 024 | How long (in minutes) do you spend per activity session in a day? | 1=less than 30 mins  2=30 mins to 60mins  3=more than 60mins |  |
| 025 | How often do you do this activity per week? | 1=Once  2=Twice  3=Three times  4=Four times  5=Daily |  |
| 026 | How do you go to school? | 1=Walking  2=Bicycle  3=Motor  4=Car |  |
| 027 | How long does it take to get to school? | …………. mins |  |
| 028 | What do you do during your leisure time? | 1=Reading  2=Watching TV  3=Playing games  4=Other (Specify)………… |  |

**Section IV: Dietary Diversity of the child**

| **S/N** | **Question** | **Responses** |  |  | **Skip to** |
| --- | --- | --- | --- | --- | --- |
| **029** | What did you eat in the last 24 hours? (Indicate details of food consumed) | **Breakfast:** ………………………………………  **Lunch:** …………………………………….  **Super:** ……………………………………... |  |  |  |
|  |  | ***Please tick as applied to the food groups*** |  |  |  |
|  | Group 1: Grains, roots and tubers | Porridge, bread, rice, noodles or other foods made from grains  White potatoes, white yams, manioc, cassava or any other foods made from roots | …….  …….. |  | ……..  ……... |
|  | Group 2:Nut and Legumes | Beans, peas, lentils, nuts or seeds | ……... |  | …….. |
|  | Group 3: Dairy products | Milk, such as tinned, powdered or fresh animal milk  Yogurt or drinking yogurt  Cheese or other dairy products | …….  ……..  …….. |  | ……..  ……..  …….. |
|  | Group 4: Flesh foods | Liver, kidney, heart or other organ meats  Beef, pork, lamb, goat, chicken or duck  Fresh or dried fish, shellfish or seafood  Grubs, snails or insects | ……….  ……….  ………..  ……….. |  | ……..  ……..  ……..  ……... |
|  | Group 5: Eggs | Eggs |  |  |  |
|  | Group 6: Vitamin A fruits and vegetables | Pumpkin, carrots, squash or sweet potatoes that are yellow or orange inside  Any dark green vegetables  Ripe mangoes (fresh or dried [not green]), ripe papayas (fresh or dried), musk melon  Foods made with red palm oil, red palm nut or red palm nut pulp sauce | …….  ……..  …….  ……. |  | …….  …….  …….  …… |
|  | Group7:Fruits and vegetables | Any other fruits or vegetables | …….. |  | ……… |
|  | Others (not counted in the dietary diversity score) | Any oil, fats, or butter or foods made with any of these  Any sugary foods, such as chocolates, sweets, candies, pastries, cakes or biscuits  Condiments for ﬂavour, such as chillies, spices, herbs or fish powder | ……..  ……..  …….. |  | ……..  ……..  ……. |

**Section V: Anthropometric Measurements**

| **S/N** | **Anthropometry** | **Responses** | **Skip to** |
| --- | --- | --- | --- |
| 030 | Weight  Weight | __________________kg  __________________kg |  |
| 031 | Height  Height | __________________m  __________________m |  |

**Section VI: Parents’ or Guardians Demographics**

| **S/N** | **Questions** | **Responses** | **Skip to** |
| --- | --- | --- | --- |
| 032A | Are you employed (mother)? | 1=Yes  2=No |  |
| 032B | What is your mother’s occupation? | 1=Student 5=Fishing  2=Housewife 6=Teacher  3=Trade/Business 7=Retired  4=Farming 8=Office Work  9=Other (Specify |  |
| 033 | Marital status | 1= Single  2=Married  3=Widowed  4=Divorced |  |
| 034 | What is the level of education of the mother? | 1=None  2=Primary  3=JSS/JHS  4=Secondary/SHS  5=Tertiary |  |
| 035 | What is the level of education of the father? | 1=None  2=Primary  3=JSS/JHS  4=Secondary/SHS  5=Tertiary |  |
| 036A | Are you employed (father)? | 1=Yes  2=No |  |
| 036B | What is the nature of work of the father? | 1=Student 5=Fishing 2=Teacher  3=Trade/Business 6=Retired  4=Farming 7=Office Work  8=Other (Specify |  |
| 037 | Average household income | GHC……………… |  |

**VII: Source of Water**

| **S/N** | **Questions** | **Responses** | **Skip to** |
| --- | --- | --- | --- |
| 039 | What is the main source of water for drinking and domestic purposes? | 1=Piped into dwelling 2=Piped into yard or plot  3=Public tap/standpipe  4=Tube well/borehole  5=Protected well 6=Unprotected well  7=Rainwater collection  8=Surface water (River, stream, lake, pond)  9=Other (Specify)…………… |  |
| 040 | What is done to the water before drinking? | 1=Nothing  2=Boiling  3=Filter  4=Refrigerate  5=Don’t Know  6=Other (Specify |  |

**VIII: Hand Washing**

| **S/N** | **Question** | **Responses** | **Skip to** |
| --- | --- | --- | --- |
| **041** | ***When do you wash your hands?*** | ***Indicate Yes or No to the statements below*** |  |
| 041A | After going to the toilet/latrine | 1=Yes  2=No |  |
| 041B | Before preparing/handling food | 1=Yes  2=No |  |
| 041C | Before feeding a child/eating | 1=Yes  2=No |  |
| 041D | After handling garbage | 1=Yes  2=No |  |
| 041E | Other (Specify) | ………………….. |  |
| **042** | ***What do you wash your hands with?*** | ***Indicate Yes or No to the statements below*** |  |
| 042A | Washes hands in a bowl of water (sharing with other people) | 1=Yes  2=No |  |
| 042B | With someone pouring a little clean water from a jug onto one’s hands | 1=Yes  2=No |  |
| 042C | Under running water | 1=Yes  2=No |  |
| 042D | Washes hands with soap or ashes | 1=Yes  2=No |  |
| 042E | Other (Specify) | ………… |  |

*THANK YOU*
